# Sex-dependent divergence in the effects of GLP-1 agonist exendin-4 on alcohol reinforcement and reinstatement in C57BL/6J mice

**DOI:** 10.1007/s00213-023-06367-x

**Published:** 2023-04-28

**Authors:** Claudia Díaz-Megido, Morgane Thomsen

**Affiliations:** 1 Laboratory of Neuropsychiatry, Psychiatric Centre Copenhagen, Mental Health Services in the Capital Region of Denmark, Copenhagen, Denmark; 2 Department of Neuroscience, Faculty of Health and Medical Sciences, University of Copenhagen, Copenhagen, Denmark

**Keywords:** Alcohol self-administration, Alcohol use disorder, Ethanol, Operant behavior, Incretin hormone, Reinstatement, Alcohol seeking, Sex differences

## Abstract

**Rationale:**

Alcohol use disorder remains a leading cause of preventable deaths, and current treatments have limited efficacy. Glucagon-like peptide 1 (GLP-1) receptor agonists can reduce alcohol drinking in preclinical studies, but mechanisms are still not fully understood, and data in female subjects are scarce.

**Objectives:**

To assess whether the GLP-1 receptor agonist exendin-4 could decrease alcohol-seeking behavior in the absence of alcohol consumption or intoxication, to compare the potency and efficacy of exendin-4 in the reduction of alcohol seeking vs. alcohol taking, and to compare effects between male and female mice.

**Methods:**

Male and female C57BL/6J mice were trained to self-administer 20% alcohol under an FR 1 schedule of reinforcement. After extinction, systemic exendin-4 (saline, 1.8, and 3.2 μg/kg) was tested in cue-induced reinstatement of alcohol seeking. Effects of exendin-4 on alcohol self-administration were tested in a separate group.

**Results:**

Exendin-4 suppressed reinstatement of alcohol seeking to extinction levels, at both doses, in the male mice, but had no effect in the female mice. Both doses of exendin-4 also significantly decreased alcohol self-administration in male mice; females again showed less pronounced effects.

**Conclusions:**

In male mice, exendin-4 appeared more effective at suppressing alcohol seeking in the absence of alcohol relative to alcohol self-administration, consistent with modulation of alcohol reward or inhibitory control, rather than satiety or aversive effects of alcohol. We saw marked sex differences with less effect of exendin-4 in female mice, and it will be important to include both sexes in further investigations into GLP-1 receptor agonists.

**Supplementary Information:**

The online version contains supplementary material available at 10.1007/s00213-023-06367-x.

## Introduction

Alcohol use disorder remains one of the top ten preventable causes of death worldwide, and, despite several medications being approved to help manage alcohol use, the condition remains severely undertreated (SAMSHA 2019). While many achieve short-term abstinence, long-term rates of relapse to harmful drinking patterns are high (about 60–80%), in both treated and untreated patients (Connor et al. 2016). According to the most recent global health data “*Over the past decade, there have been particularly large and concerning increases […] in exposure to several highly preventable risks—obesity, high blood sugar, alcohol use, and drug use*” (healthdata.org and (Vos et al. 2020). GLP-1 receptor agonists are medications approved to treat both type 2 diabetes and obesity (Courtney et al. 2017; Dar et al. 2015; Drucker et al. 2017; Eng et al. 2014; Pi-Sunyer et al. 2015), and show promise in laboratory animal studies as treatments to reduce alcohol drinking as well as other substance use disorders (for review, see Eren-Yazicioglu et al. 2020; Klausen et al. 2022a; Shevchouk et al. 2021). Since GLP-1 systems modulate both energy homeostasis, consummatory behaviors, and non-food (drug) reward, it remains unclear how much of the reduction of alcohol drinking relies on the availability of alcohol, and mechanisms such as satiety or aversion. A recent double-blind placebo-controlled 26-week clinical trial failed to detect an effect of depot formulation exendin-4 on drinking measures in treatment-seeking patients with alcohol use disorder, relative to placebo, although effects seemed to vary by body mass index (Klausen et al. 2022b). In contrast, the same study did find that exendin-4 treatment reduced alcohol cue reactivity measured by fMRI (Klausen et al. 2022b), which has been shown to predict drug or alcohol use (Vafaie and Kober 2022). These findings further raise questions about how GLP-1 receptor agonists modulate alcohol-related effects such as consumption, reward, and craving.

Drug self-administration studies commonly use reinstatement paradigms to differentiate between drug-seeking behaviors (in the absence of drug) and drug-taking behaviors. GLP-1 receptor agonists have been shown to reduce or prevent reinstatement of cocaine or heroin seeking, using assays of cue- or drug-induced reinstatement of an operant response previously reinforced by drug infusions (Douton et al. 2021; Douton et al. 2022; Hernandez et al. 2018). Reinstatement of alcohol-seeking operant behavior is increasingly employed, with stress or cue-induced reinstatement of alcohol seeking being demonstrated in rats trained to self-administer alcohol both orally and intravenously (Gass and Olive 2007; Gass et al. 2014; Zhao et al. 2006). Fewer studies have used mice, but cue-induced reinstatement of alcohol seeking has been shown in male mice trained to self-administer alcohol orally (Nuutinen et al. 2016; Salling et al. 2017; Sanchis-Segura et al. 2006; Walker et al. 2015). Importantly, effects of GLP-1 receptor agonists on reinstatement of operant alcohol seeking have not been tested.

Also, few studies have compared the efficacy of GLP-1 receptor agonists on alcohol-related behaviors between male and female subjects, but studies on food reinforcement suggest sex differences (Lopez-Ferreras et al. 2019; Lopez-Ferreras et al. 2018; Richard et al. 2016). GLP-1 receptor antagonist (exendin-9) administration or GLP-1 receptor knockdown also showed diverging effects on sucrose reinforcement between male and female rats (Lopez-Ferreras et al. 2018; Maske et al. 2018). The long-acting GLP-1 receptor agonist dulaglutide decreased alcohol drinking in both male and female rats using a two-bottle intermittent access assay, both acutely and as sustained treatment (Vallöf et al. 2020). However, the males showed a more robust reduction and showed some residual reduction in alcohol intake, relative to vehicle controls, after discontinuation of dulaglutide treatment, while females resumed vehicle-level drinking essentially immediately upon treatment cessation (Vallöf et al. 2020). Finally, GLP-1 receptor polymorphisms were found to be associated with alcohol use disorder, but the association was more robust in men than in women (Suchankova et al. 2015), consistent with the possibility that GLP-1 receptor function may modulate effects of alcohol differentially between men and women.

Here, we investigated whether exendin-4 pretreatment could reduce cue-induced reinstatement of an operant response previously reinforced with alcohol, in male and female mice. To compare this effect of exendin-4 in the absence of alcohol to the suppression of alcohol-taking behavior, we also tested exendin-4 in alcohol self-administration.

## Methods

### Subjects

We report data from 34 male and 39 female C57BL/6JRj (Janvier, France) mice, acquired at 6 weeks of age and acclimated 1 week to the facilities before testing. Mice were housed in same-sex groups 4–6 per cage with hiding devices/handling tube, nesting material, climbing rope, and wooden chewing block as enrichment, under a reversed light-dark cycle (light on 19:00 to 07:00) in a temperature- and humidity-controlled room. Microchips (UNO Micro ID-12) were implanted subcutaneously under brief sevoflurane anesthesia for unambiguous identification. Procedures conformed to international ethics standards and were approved by the Animal Experiments Inspectorate under the Danish Ministry of Food, Agriculture, and Fisheries in accordance with the EU directive 2010/63/EU for animal experiments. Tap water and standard rodent chow (Altromin 1324) were freely available in the home cages. Testing occurred during the dark phase, Monday–Friday. In an effort to follow the 3R principles, as a secondary goal, we tried testing reinstatement with vehicle and drug within-subjects. The order of testing significantly affected reinstatement (but not alcohol self-administration); therefore, only data from the first reinstatement test were used.

### Experimental design

**Fig. 1:**
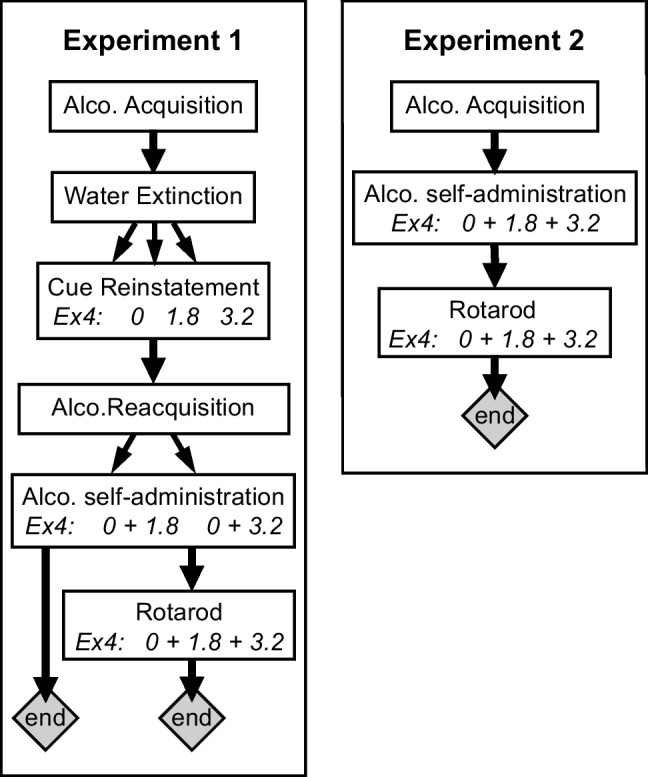
Diagram of experimental design. The sequence of training and testing is shown for the original experiment (experiment 1) and for the replication of the alcohol self-administration part (experiment 2). Male and female mice were tested concurrently in both experiments. Acquisition, extinction, and reacquisition phases lasted until preset criteria were met, with each mouse progressing to the next step as they met criteria. Alco, alcohol; Ex4, exendin-4 (or saline) pretreatment. In experiment 1, exendin-4 doses were tested between subjects or one dose + saline, shown as splitting arrows. In experiment 2, since there was no reinstatement (which was tested only once), exendin-4 doses were tested within-subjects (no splitting arrows)

In experiment 1, male and female mice were trained in operant-conditioning chambers to self-administer alcohol orally (typically 15–20 sessions); then, responding was extinguished to criteria by replacing alcohol with water (2 to 20+ sessions), and cue-induced reinstatement of nose-poking was tested with exendin-4 or saline pretreatment, between subjects. Then, alcohol was again made available to allow mice to re-acquire self-administration, and the effect of exendin-4 pretreatment on alcohol self-administration was tested (see Supplemental Figure S1). Because re-acquisition of alcohol was variable and some mice decreased responding after saline injection following the reinstatement test, we performed a second experiment (experiment 2) in drug-naïve mice, in which the effect of exendin-4 pretreatment on alcohol self-administration was assessed without prior testing. These, and a subset of experiment 1 mice, were tested in the rotarod assay after ended operant testing. Figure 1 shows a diagram of the experimental design.

### Operant-conditioning apparatus and training

Standard mouse operant-conditioning chambers (Med-Associates, St. Albans) enclosed in sound-attenuating cubicles were used, each equipped with a fan, house light (light level in chambers when on: ~ 25 lux; Fisherbrand Traceable dual-range light meter, Traceable, Webster, TX, USA), two nose-poke holes (one active, one inactive), and a steel cup for liquid reinforcers delivery, as previously described (Bornebusch et al. 2019). Mice were allowed to acquire nose-poking under a fixed ratio 1 timeout 20-s schedule of reinforcement in daily 2-h sessions, first with a non-sweet liquid food (Nutridrink, Vanilla flavor, Nutricia) as the reinforcer. A yellow cue light was turned on for 20 s along with each reinforcer delivery. When at least 20 reinforcers were earned per session for two sessions (typically 2–4 sessions), food was replaced with the oil-water emulsion Calogen (unflavored, Nutricia), then 10% alcohol in Calogen, then 20% alcohol in Calogen, for at least two sessions each and until at least 15 reinforcers were earned (typically 2 sessions each). Liquid reinforcers were 30 μl. Finally, responses were reinforced with 20% alcohol in water for a minimum of five baseline sessions and throughout testing. Mice met baseline criteria when they maintained ≥ 70% responses in the active nose-poke hole and a ≥ 15/session reinforcer average over five consecutive sessions, with one session > 10 but < 15 reinforcers acceptable if followed by at least two sessions ≥ 15. Fifteen reinforcers/session equates to 3.0 g/kg alcohol for a 24-g mouse. Any alcohol solution left unconsumed at the end of the session was measured using a pipette (typically none or very little), and the estimated alcohol intake as g/kg was calculated. A limit of 50 alcohol reinforcers per session (9.9 g/kg in a 24-g mouse) was implemented to prevent overdose (Sørensen et al. (2016) and unpublished observations that many female C57BL/6JRj mice drank to unresponsiveness (“passing out”) if left unlimited in the current assay). The average reinforcers taken over the last two sessions was used as baseline level. In experiment 1, once self-administration criteria were met, alcohol solution was replaced with water and the alcohol-associated cue light remained off, all other parameters remaining constant. Extinction criteria were met when responding in the active nose-poke hole reached < 30% of baseline level.

### Testing

Experiment 1: Once extinction criteria were met, mice were tested for cue-induced reinstatement of alcohol seeking in the following session, except that tests never occurred on a Monday (if extinction criteria were met on a Friday, an additional extinction session was given on the following Monday, or more until extinction criteria were again satisfied). Reinstatement sessions were identical to baseline sessions, except that water, not alcohol, was delivered. Mice were assigned to test either saline, 1.8 μg/kg exendin-4, or 3.2 μg/kg exendin-4. Mice were tested in four successive cohorts and were assigned following an alternation pattern in the order that they met criteria so that all treatment groups were tested in parallel, balanced across time. An exception to this was the addition of a fifth cohort to add mice to the male 3.2 μg/kg group (plus saline controls) to rectify a too-low group size. Mice were weighed immediately before the injection and 24 h later, to test for acute effects on bodyweight.

Experiment 2: Once baseline criteria were met, mice were given a saline injection 30 min before the session as habituation; if reinforcers earned dropped below the range of reinforcers earned during the baseline sessions, a second habituation was given once self-administration restabilized. Then, saline, 1.8, or 3.2 μg/kg exendin-4 were tested within subjects in a predetermined, counterbalanced sequence. Because the previous repeated tests (supplemental Fig. S2) suggested a brief carry-over effect of exendin-4 administration, mice were given at least 6 days between administration of exendin-4 and the next test, during which baseline sessions were continued.

### Motor function control test

Mice in the fourth cohort of experiment 1 (8 males and 8 females) and mice in experiment 2 (10 males and 9 females) were tested on an accelerating rotarod (Ugo Basile Biological Research Apparatus Model 7650, Varese, Italy) after completion of the operant experiments. Speed increased from 4 to 40 rpm over 5 min, then, remained at 40 rpm. Time to fall off (or stay on without walking for one rotation) was recorded. The testing room was set to low illumination (~ 45 lux). In experiment 1, mice received saline, 1.8, or 3.2 μg/kg exendin-4 30 min before the test, in the same sequence as they received it before, the dose they had not previously experienced being tested last. This yielded high variability (not shown). Therefore, in experiment 2, mice were tested at least 1 week after completing operant sessions, and they received a training session 1 day before they started testing. Then, doses were tested in ascending order, with 2 days off between 1.8 and 3.2 μg/kg exendin-4 to limit possible carryover effects.

### Drugs

Alcohol (undenatured ethanol 96%, Plum A/S, Denmark) was diluted to 20% v/v in tap water or to 10 and 20% in Calogen. Frozen lyophilized exendin-4 (Bio-Techne, UK) was dissolved in 0.9% saline on ice, and aliquots were kept at − 18 °C; a fresh aliquot was defrosted and diluted to the desired concentration each test day. Saline and exendin-4 were administered by 10 mL/kg intraperitoneal injections, 30 min before test sessions.

### Data analysis

Statistical analyses were performed with InVivoStat software v. 4.6 and StataSE v. 13.1; *p* values < 0.05 are reported as significant according to convention, but all statistical outcomes are reported, regardless of this arbitrary cutoff value. Data are presented as individual points and/or as group mean ± SEM.

Reinstatement tests: We first analyzed whether cue presentation did reinstate nose-poking in the previously alcohol-reinforced nose-poke hole. Liquid deliveries (alcohol or water “reinforcers”) were compared in each sex by two-way ANOVA with condition (baseline/extinction/reinstatement) as repeated-measure factor and treatment group as between-subject factor, with cohort as a blocking (random) factor. Significant main treatment effect or treatment by condition interaction was followed by one-way repeated-measure ANOVA in each treatment group and paired-sample *t* test of extinction vs. reinstatement, corrected for false discovery rate (within each sex = three comparisons, Benjamini-Hochberg procedure, 5% limit). Numbers of nose-pokes in the inactive hole were analyzed in the same way.

To directly assess sex differences, responses in the active hole during reinstatement (i.e., cue + water deliveries) were then compared by two-way ANOVA with sex and treatment group as between-subject factors, with cohort as a blocking factor. Any significant main effect or interaction was followed by one-way ANOVA in each sex and multiple pair-wise comparisons vs. saline (Benjamini-Hochberg procedure). Inactive responses were analyzed in the same way. Alcohol reinforcers earned, as well as inactive responses, in the self-administration test (experiment 2) were similarly analyzed by two-way ANOVAs with sex as between-subject factor and exendin-4 dose as repeated-measure factor. Intraperitoneally administered exendin-4 has an estimated half-life approximately 2.5 h in male rats, and maximal blood glucose-lowering effects of 1 to 4 μg/kg exendin-4 lasted for at least 4 h in male mice, which is longer than the 2-h session plus 30-min pretreatment time used here (Young et al. 1999; Parkes et al. 2001). However, reports comparing the pharmacokinetics of exendin-4 between male and female rodents are lacking. Therefore, we also analyzed data separately for the first and second hours of the session. Reinstatement data were also analyzed as total responses in the active nose-poke hole including timeout responses, but these analyses did not provide any additional information compared to the data without timeout responses and are therefore not reported for brevity.

As secondary measures, alcohol reinforcers earned and estimated alcohol intake during baseline sessions were compared between sexes by two-way ANOVA with session as repeated-measure factor and sex as between-subject factor, blocked by cohort. Water deliveries during extinction could not readily be analyzed by ANOVA since mice took variable numbers of sessions to meet criteria, and then proceeded to reinstatement testing. Rather, sessions needed to achieve extinction criteria were analyzed by log rank test with sex as factor (and cohort as blocking factor); in addition, responses in the active hole as a function of extinction session were fitted by simple linear regression. We also compared treatment groups and confirmed that groups that went on to test saline, 1.8, and 3.2 μg/kg exendin-4 were equivalent during both baseline and extinction phases (*p* ≥ 0.4 main effect and interactions). Since mice had differing numbers of extinction sessions, we also verified that responding at extinction criteria and in the reinstatement tests was not significantly related to the number of extinction sessions (*p* ≥ 0.3). Latencies to fall from the rotarod were analyzed by Cox proportional hazard regression with sex and exendin-4 dose as factors. Bodyweight changes (day-after minus pre-injection) were comparable between sexes and are therefore reported for sexes combined.

## Results

### Baselines and extinction (experiment 1)

**Fig. 2:**
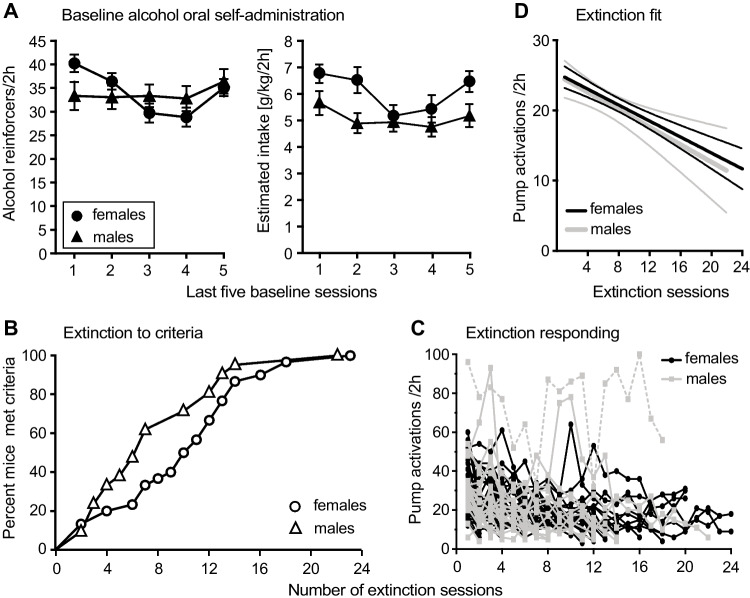
Baseline and extinction in female and male mice, experiment 1. **A** Alcohol reinforcers earned and estimated alcohol intake (g/kg) per 2-h session in female (circles, *n* = 35) and male (triangles, *n* = 26) mice in the last five baseline sessions, as a function of successive session number. **B** Percentage of mice meeting extinction criteria as a function of number of extinction sessions. **C** Water deliveries earned per 2-h session in individual mice as a function of extinction sessions, one outlier shown with dashed line. **D** Linear regression of extinction in female and male mice. Data are group means ± SEM (**A**), individual mice (**B**, **C**), or best fit with 95% confidence limits (**D**)

Figure 2 shows the baseline alcohol self-administration and extinction during water substitution in male and female mice. Alcohol reinforcers earned and estimated alcohol intake at baseline varied by day ([*F*(4, 236) = 6.36, *p* < 0.0001], [*F*(4, 236) = 5.25, *p* = 0.0005]), with no effect of sex (*p* = 0.6 and *p* = 0.3). There was a trend for sex by day interaction for reinforcers earned [*F*(4, 236) = 2.41, *p* = 0.050], which was not maintained in the estimated alcohol intake corrected for bodyweight (*p* = 0.2). While self-administration seemed more stable in males than in females, differences were not significant post hoc on individual days (Fig. 2A). Experiment 2 showed no significant effect of time, sex, or interaction in the baseline measures (*p =* 0.2−0.5, data not shown).

For extinction, survival analysis showed no significant sex difference in latency to extinction criteria (Fig. 2B, *p =* 0.09). Responding in the active nose-poke hole for each mouse is shown in Fig. 2C. Linear regression showed comparable lines for females and males (slopes − 0.57 and − 0.62, respectively, *p =* 0.8; *Y* intercept 25.3 and 25.1, *X* intercept 44.6 and 40.42, *p =* 0.5; Fig. 2D) after exclusion of one male outlier that showed very high responding throughout (with the outlier included, slope was flat and the confidence interval for intercept was 50.4 to infinity). Five out of 35 female mice and two out of 26 male mice failed to meet extinction criteria and were excluded from further testing after 19–23 sessions with no decreasing trend in responding at that time.

### Cue-induced reinstatement of alcohol seeking (experiment 1)

**Fig. 3:**
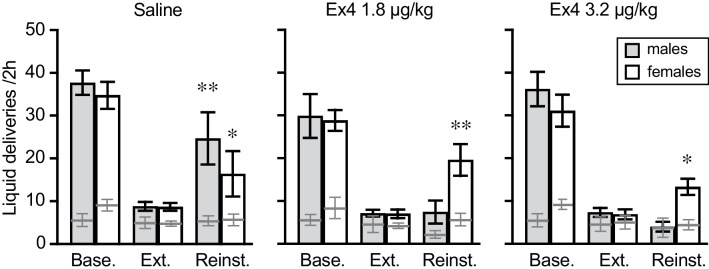
Acute effect of exendin-4 on cue-induced reinstatement of alcohol seeking, experiment 1. Alcohol reinforcers earned and water deliveries per 2-h session in female (*n* = 10/dose group) and male (*n* = 6-11/dose group) mice under conditions of alcohol availability (baseline, “Base.”), at extinction criteria with responding resulting in water delivery and no light cue (“Ext.”), and during the reinstatement test with response-contingent cue presentation and water delivery (“Reinst.”). Responses made in the inactive nose-poke hole are shown as dark grey lines. All data are groups means ± SEM. *p < 0.05, **p < 0.001 vs. extinction

The female mice showed nose-poking behavior related to the condition (baseline, extinction, or reinstatement test) [*F*(2, 54) = 64.6, *p* < 0.0001], but showed no effect of exendin-4 treatment or treatment by condition interaction (*p* > 0.4; Fig. 3). Removal of one outlying value at > 4 standard residuals to conform to normality assumptions did not reveal or remove any significant findings. Planned post hoc tests showed significantly higher responding in the reinstatement test relative to extinction in all three dose groups (see Fig. 3). Responses in the inactive nose-poke hole decreased between baseline criteria and extinction criteria as revealed by a significant effect of condition [*F*(2, 52) = 11.5, *p* < 0.0001], regardless of exendin-4 treatment group or interactions (*p* > 0.5). Scrutiny of the main effect post hoc confirmed significantly lower inactive responses at extinction and reinstatement relative to the baseline condition (both *p* < 0.0001) but no effect of the reinstatement test relative to extinction (*p* > 0.8).

In contrast, in the male mice, nose-poking was related to condition [*F*(2, 42) = 70.8, *p* < 0.0001] with a significant treatment by condition interaction [*F*(4, 42) = 4.15, *p =* 0.006]. Again, removal of one statistical outlier to conform to normality assumptions did not change these conclusions. There was a marked effect of test condition in all three groups of male mice (all *p* < 0.0001), but only the saline-treated group showed cue-induced reinstatement of alcohol seeking as defined by significant increase in water deliveries in the reinstatement test relative to the extinction condition (saline *p =* 0.002, or *p =* 0.0004 with outlier removed; 1.8 μg/kg exendin-4 *p =* 0.94, 3.2 μg/kg exendin-4 *p =* 0.35; Fig. 3). Inactive responses in male mice showed no effect of condition, group, or interaction (*p* > 0.3). Thus, female mice showed reinstatement of alcohol seeking regardless of exendin-4 treatment, whereas exendin-4 blocked reinstatement in male mice.

Comparison of reinstatement of nose-poking between male and female mice that received saline confirmed an effect of test [*F*(1, 38) = 45.2, *p* < 0.0001] but showed no significant effect of sex or sex by test interaction (*p* > 0.4). Post hoc test confirmed higher responding during the reinstatement test than under extinction (*p =* 0.004). Inactive responses were not significantly related to either sex or condition (all *p =* 0.2).

**Fig. 4:**
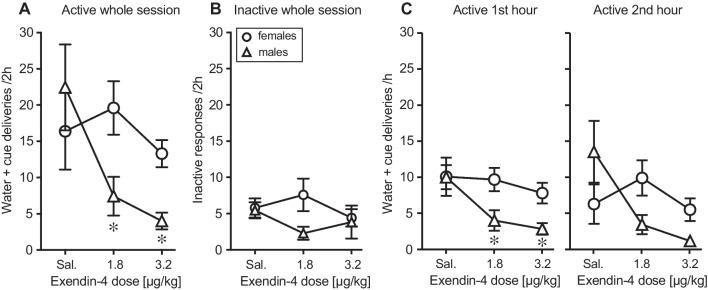
Dose-response relationship of acute exendin-4 in cue-induced reinstatement of alcohol seeking, experiment 1. Water + cue deliveries per 2-h session (**A**) and nose pokes in the inactive hole per session (**B**) during cue-induced reinstatement in female mice (circles, *n* = 10) and male mice (triangles, *n* = 6–11) as a function of exendin-4 pretreatment dose. **C** Water + cue deliveries in the first and second hours of the reinstatement session in female and male mice as a function of exendin-4 pretreatment dose. Data are the same as in Fig. 3 and are group means ± SEM. *p < 0.05 vs. saline

Analysis of responding in the previously alcohol-reinforced nose-poke hole during the cue-induced reinstatement test (Fig. 4A) revealed significant effects of exendin-4 dose [*F*(2, 43) = 5.20, *p =* 0.01] as well as sex-by-dose interaction [*F*(2, 43) = 4.87, *p =* 0.01], while the main effect of sex did not quite reach significance [*F*(1, 43) = 3.84, *p =* 0.056]. Analysis of the dose effect in each sex revealed the interaction to be due to male mice showing an effect of exendin-4 [*F*(2, 16) = 4.57, *p =* 0.03] whereas the female mice did not (*p* > 0.5). Both doses of exendin-4 decreased reinstatement responding in the male mice (*p* < 0.05 vs. saline). Responses in the inactive hole were not significantly related to sex, dose, or sex-by-dose interaction (*p* > 0.4; Fig. 4B).

Due to the relatively short half-life of exendin-4 in rodents and to allow for possible, as yet unidentified, pharmacokinetic differences of exendin-4 between the sexes, active responses were also analyzed separately for the first and second hours of the reinstatement session (Fig. 4C). In the first hour, responding was related to exendin-4 dose [*F*(2, 42) = 5.87, *p =* 0.006] with near-significant effects of sex [*F*(2, 42) = 4.04, *p =* 0.051] and sex-by-dose interaction [F(2,42) = 3.09, *p =* 0.056]. As with the full session, responding decreased as a function of exendin-4 dose in the males [*F*(2, 15) = 6.07, *p =* 0.01] but not in the females (*p =* 0.8). In the male mice, both doses of exendin-4 suppressed reinstatement relative to saline (*p =* 0.04). During the second half of the session, only the sex-by-dose interaction was significant (*p =* 0.02). Although the effect of exendin-4 was still apparent in the males only, there was more variation, and the dose effect did not reach significance (*p =* 0.09).

Mice were weighed before each injection and again 24 h later and showed no acute effect of exendin-4 administration on bodyweight during reinstatement tests: saline 0.04 ± 0.07 g, exendin-4 1.8 μg/kg − 0.16 ± 0.07 g, exendin-4 3.2 μg/kg − 0.11 ± 0.09 g (bodyweight difference, day-after minus pre-injection).

### Oral alcohol self-administration (experiment 2)

**Fig. 5:**
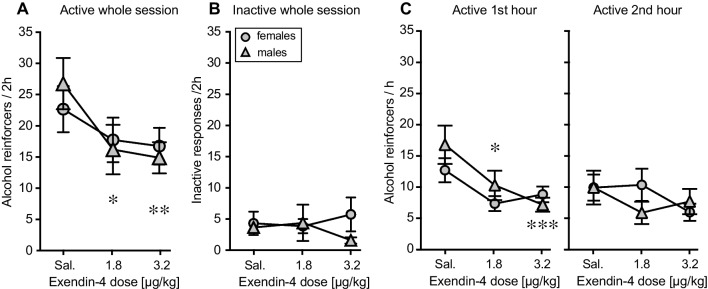
Acute effect of exendin-4 on alcohol self-administration, experiment 2. Alcohol reinforcers per 2-h session (**A**) and nose pokes in the inactive hole per session (**B**) in female mice (circles, *n* = 8–9) and male mice (triangles, *n* = 10) as a function of exendin-4 pretreatment dose. **C** Alcohol reinforcers in the first and second hours of session in female and male mice as a function of exendin-4 pretreatment dose. Data are group means ± SEM. *p < 0.05, **p < 0.001, ***p < 0.0001 vs. saline

Analysis of alcohol reinforcers taken showed an effect of exendin-4 dose [*F*(2, 32) = 6.52, *p =* 0.004] but no significant effect of sex (*p =* 0.90) or interaction (*p =* 0.53) despite an apparent flatter curve in the females than in the males (Fig. 5A). Planned follow-up analysis in each sex showed that exendin-4 dose significantly reduced alcohol self-administration in the males [*F*(2, 18) = 5.80, *p =* 0.01], with both doses reaching significance relative to saline (*p =* 0.01, *p =* 0.006) while the dose effect did not reach significance in the females (*p =* 0.25). Responses in the inactive hole were not significantly related to sex, dose, or sex-by-dose interaction (*p* > 0.36; Fig. 5B).

Self-administration data were also analyzed in the first and second hours to account for possible sex differences in pharmacokinetics. Exendin-4 had clear effects in the first hour [*F*(2, 31) = 9.75, *p =* 0.0005] (Fig. 5C), with no significant effect of sex (*p =* 0.43) or interaction (*p =* 0.21). Analysis in each sex revealed a significant exendin-4 effect in the males [*F*(2, 18) = 8.13, *p =* 0.003], with both doses decreasing self-administration (*p =* 0.02, *p =* 0.0009 vs. saline). The effect approached significance in the females (*p =* 0.08), with no dose reaching significant after multiple comparison adjustments. In the second hour, there were no significant effects of any factor (Fig. 5C; *p =* 0.26−0.70).

We verified that baseline levels before each test day were comparable across doses and with no dose-by-sex interaction (see Supplemental Figure S2; *p* > 0.7), although females tended to take fewer reinforcers (*p =* 0.08). The baseline day immediately after testing suggested some carryover effect of exendin-4 with a trend for dose effect [*F*(2, 32) = 2.50, *p =* 0.098], regardless of sex (main effect and interaction *p* > 0.8).

### Motor function control test

**Fig. 6:**
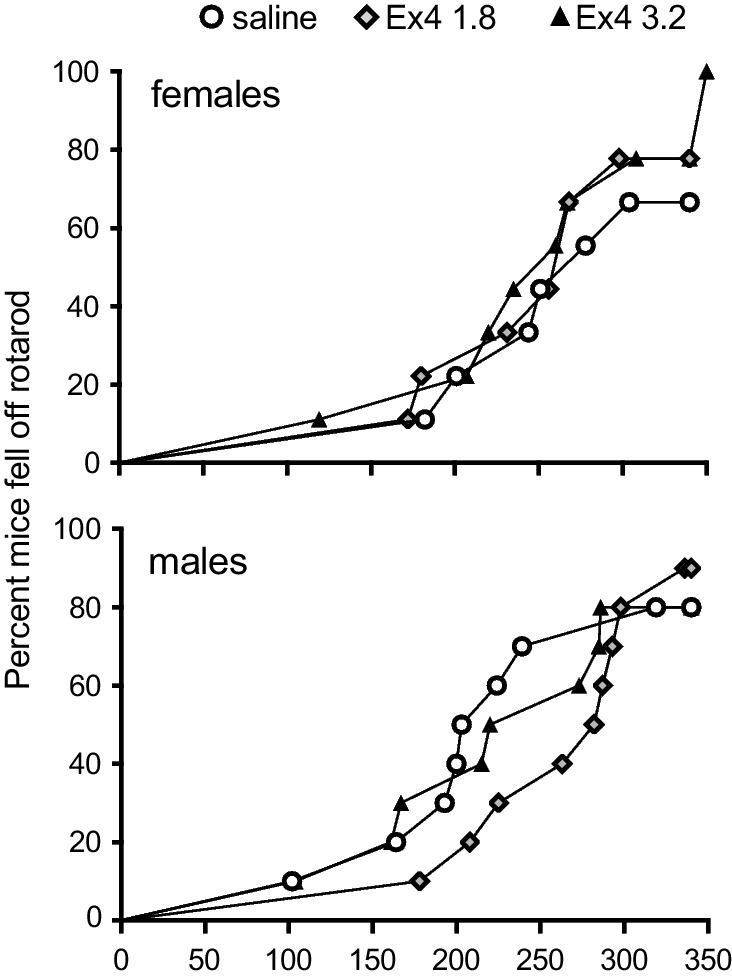
Acute effects of exendin-4 in the rotarod motor performance test, experiment 2. Proportion of female and male mice falling off the accelerating rotarod as a function of testing time (seconds) after pretreatment with saline or 1.8 or 3.2 μg/kg exendin-4. *n* = 8–10. Data points represent individual mice

Because male mice treated with 3.2 μg/kg exendin-4 during reinstatement testing showed very low levels of responding, we used an accelerating rotarod assay to test for gross motor impairments (Fig. 6). Cox regression showed no significant effect of sex (*p =* 0.7) or of exendin-4 dose (*p =* 0.5).

## Discussion

We found that systemic, acute administration of 1.8 and 3.2 μg/kg exendin-4 decreased oral alcohol self-administration in male mice, consistent with previous findings with oral and intravenous alcohol self-administration in male rats and mice under various schedules of reinforcement (Sørensen et al. 2016; Bornebusch et al. 2019). Further, both doses of exendin-4 completely prevented reinstatement of alcohol-seeking behavior in the male mice taken as a group mean (same numbers of responses during extinction and reinstatement). Looking at individual mice, only one male mouse increased responding during the reinstatement test in the 1.8 μg/kg group, none in the 3.2 μg/kg group. Although we did not compare the two different tests statistically, the magnitude of reduction appeared greater in the reinstatement test than in the self-administration test (near-100% reduction vs. < 50% reduction). In other words, exendin-4 may be more potent and/or more effective in reducing alcohol seeking in the absence of the reinforcer than at reducing alcohol intake, at least in male mice. This result is consistent with a recent clinical trial finding reduced alcohol cue reactivity measured by fMRI after exendin-4 treatment despite a lack of effect on drinking measures (Klausen et al. 2022b). At the doses tested here, exendin-4 did not impair motor performance on the accelerating rotarod. Further, responding in the non-reinforced (inactive) nose-poke hole was not significantly affected by exendin-4. Thus, the decreases in operant behaviors were not likely due to reduced ability to perform the task.

Surprisingly, we found that exendin-4 was ineffective at reducing reinstatement of alcohol seeking in the female mice and showed a less robust effect on alcohol self-administration in the female mice than in the male mice. Indeed, significant decreases in self-administration were also only obtained in the males, though the clear trend in effect and lack of overall sex difference suggests that a larger sample size would have yielded statistical significance in both sexes. Higher doses of exendin-4 are expected to significantly suppress psychomotor activity (Krass et al. 2012; Sørensen et al. 2015; Bornebusch et al. 2019), although we cannot exclude that female mice may show a reduction in alcohol-seeking at a higher dose. In an initial test in the mice that had already tested reinstatement effects, females seemed more affected by the injections per se, as they self-administered less alcohol after saline injections than at baseline, which could have contributed to masking a possible effect of exendin-4. However, in a group of experimentally naïve mice, this was not the case, but still exendin-4 did not decrease alcohol self-administration as clearly in the females as in the males. The female mice appeared to show less robust reinstatement of alcohol seeking relative to the males (in the saline groups) over the whole session. However, when looking at the first hour only, saline-treated males and females made equal numbers of alcohol-seeking responses and exendin-4 robustly and dose-dependently decreased seeking in the males but not in the females, indicating that the lack of effect in the females cannot be attributed to a “floor effect.” Thus, female mice showed blunted effects of exendin-4 on alcohol seeking and self-administration.

Reasons for this sex difference remain to be elucidated. One possibility is that GLP-1 receptor expression could differ between sexes. Few reports have addressed this, but some findings suggest moderately differential expression, such as higher expression in nucleus accumbens of male rats than of female rats (Lopez-Ferreras et al. 2019). Sex differences in electrophysiological properties of GLP-1 neurons have also been reported (Zeng et al. 2021), although their physiological significance in relation to the present findings is unclear. Alternatively, there may be interactions between gonadal hormones and GLP-1 receptor-mediated effects. Indeed, some effects of GLP-1 receptor agonists (e.g., food reward, weight loss) were dependent on, or potentiated by, estrogens in female rodents (Asarian et al. 2012; Richard et al. 2016; Maske et al. 2017). Conversely, GLP-1 receptor agonists were shown to affect gonadal function in female rodents (Outeiriño-Iglesias et al. 2015; Saber and Abd El-Rahman 2019). How these interactions may explain the reduced effects of exendin-4 in the present study is also unclear.

Intra-cerebroventricular exendin-4 administration suppressed sucrose oral self-administration (under a progressive ratio schedule of reinforcement) in female rats but had less effect in male rats (Richard et al. 2016). When authors from the same group administered exendin-4 specifically into the lateral hypothalamus, they found the converse, that male rats showed greater reduction in sucrose self-administration than female rats (Lopez-Ferreras et al. 2018). Further, GLP-1 receptor knockdown or antagonist administration increased sucrose self-administration, bodyweight, feeding, and fat in adult male rats but not in adult female rats (Lopez-Ferreras et al. 2018). Pretreatment with the GLP-1 receptor antagonist exendin-9 similarly prevented intragastric pre-feeding from suppressing operant sucrose self-administration in male but not female rats (Maske et al. 2018). In the same studies, exendin-4 appeared to suppress free peanut butter and free chow intake comparably in both sexes (Lopez-Ferreras et al. 2018; Richard et al. 2016). Similarly for the ventral tegmental area (VTA) and the supramammillary nucleus, effects of targeted microinfusion of exendin-4 were sexually divergent on operant assays of sucrose reinforcement but not in consumption of chow, sucrose, or other palatable food (Lopez-Ferreras et al. 2019). Taken together with our observation that female mice showed less effects of exendin-4 in both reinstatement of alcohol seeking and in alcohol self-administration, those findings suggest that male and female rodents may differ in how GLP-1 receptor agonists modulate reward or operant performance, rather than in consummatory behaviors or satiety. However, dulaglutide treatment was more effective at reducing alcohol intake in male than in female rats using a two-bottle assay of intermittent alcohol access assay (Vallöf et al. 2020), indicating that the lesser effectiveness of GLP-1 receptor agonists to reduce alcohol reward in female rodents, relative to males, extends beyond operant self-administration conditions. This would seem to represent a difference between the modulation of rewarding effects of food or sucrose vs. alcohol (sex difference observed only under operant conditions), perhaps because alcohol-taking behaviors are more dependent on reward pathways and less on satiety and calory intake regulation mechanisms, relative to food, although this interpretation is speculative.

Those different outcomes in sex differences when using operant alcohol self-administration vs. home-cage alcohol access are reminiscent of findings reported using quinine as “punisher,” an aversive taste that can reduce alcohol taking. Indeed, using a modified drinking in the dark 2-bottle choice procedure in C57BL/6 mice, both sexes showed comparable aversion-resistant drinking during adulteration with quinine (Sneddon et al. 2019). In contrast, using an operant oral alcohol self-administration assay, the same research group observed that female mice maintained alcohol self-administration at quinine concentrations that markedly suppressed responding in the male mice, despite equal sensitivity to quinine avoidance (in water bottles) in the home cage and equal sensitivity in quinine-suppressed sucrose self-administration (Sneddon et al. 2020). Thus, if GLP-1 reduces alcohol intake by producing or enhancing aversive effects of alcohol, sex differences in the efficacy of aversion to reduce alcohol intake may participate in the differences we observed in the present study. However, aversion caused by a tastant like quinine and aversion caused by exendin-4 (e.g., nausea) may not rely on the same mechanisms. In alcohol-preferring P rats self-administering alcohol under a fixed or progressive ratio schedule of reinforcement, females showed less decrease in alcohol self-administration than males after acute pretreatment with topiramate, naltrexone (trend), or a topiramate + naltrexone combination (Moore and Lynch 2015). Whatever the mechanism, female rodents may be more resistant to manipulations that reduce alcohol self-administration in general, not only GLP-1 receptor agonists. Oxytocin decreased alcohol drinking in male but not female prairies voles (Potretzke et al. 2022), although a study in mice found similar or higher reduction in stressor-induced reinstatement of alcohol seeking after oxytocin administration in female mice (King and Becker 2019), suggesting sex differences vary by experimental conditions and pharmacological target.

In addition to possible differences in sensitivity to GLP-1 receptor agonists, rodent studies have shown sex differences in alcohol-drinking behaviors. We found no significant differences between male and female mice in alcohol self-administration, extinction of nose-poking behavior, or reinstatement of alcohol seeking, consistent with some previous reports in mice (Jensen et al. 2021; King and Becker 2019). Female rats and mice often show larger alcohol intakes than males in home-cage bottle drinking assays, but sex differences are less consistent for operant alcohol self-administration, in which males and females often earn comparable numbers of alcohol reinforcers (e.g., Hernandez et al. 2020; Moore and Lynch 2015; Sneddon et al. 2020; Sneddon et al. 2019; Tambour et al. 2008; Zhou et al. 2017; and see Becker and Koob 2016 for review). It has also been suggested that in humans and in rodents, females may be more sensitive to the effect of stressors to promote alcohol drinking (see review by Mineur et al. 2022). Still relatively few studies have compared alcohol reinstatement between males and females. Some studies found that female rats are more sensitive than the males to reinstatement of alcohol-seeking behavior by alcohol-associated cues or stressors (Bertholomey et al. 2016; Surakka et al. 2021); other showed no sex differences (Hernandez et al. 2020; King and Becker 2019), perhaps due to methodology varying between investigations.

To conclude, our study suggests that exendin-4 is less effective at decreasing alcohol-taking behavior compared to alcohol seeking in the absence of alcohol. This finding is consistent with GLP-1 receptor agonists modulating rewarding effects of alcohol or inhibitory control, and not (or less so), by the combination of alcohol and exendin-4 producing satiety or aversive effects. In this sense, the present findings are compatible with recent studies which proposed that GLP-1 receptor activation modulates inhibitory control of responding (for food reward) rather than appetitive drive (Hsu et al. 2018; Jones et al. 2019). Few studies on GLP-1 receptor agonist modulation of alcohol effects have so far included both sexes, and our findings suggest that exendin-4 treatment may be less effective or less potent in females than in males. This finding is consistent with a previous study using liraglutide in rats (Vallöf et al. 2020), and our study shows an even larger sex difference for alcohol seeking than for alcohol drinking or self-administration.

## Supplementary information

ESM 1: (PDF 135 kb)
