# Supplementary material for: Sex-dependent divergence in the effects of GLP-1 agonist exendin-4 on alcohol reinforcement and reinstatement in C57BL/6J mice
Source: Psychopharmacology (Berl). 2023 Apr 28;240(6):1287–98. doi: 10.1007/s00213-023-06367-x (PMC10172234; doi:10.1007/s00213-023-06367-x)
Supplement: Supplementary file 1 — (PDF 135 kb) [file 213_2023_6367_MOESM1_ESM.pdf]

Sex differences in the effects of GLP-1 agonist exendin-4 on alcohol reinforcement and reinstatement in C57BL/6J mice

Claudia Díaz-Megido and Morgane Thomsen

Supplemental materials

Reinstatement test within-subjects (Experiment 1)

In an effort to follow the 3R principles, we tried testing vehicle and drug within-subjects, challenging the convention that reinstatement tests are most typically only done once. Our "backup strategy" was to use only the first test in each animal and add more subjects if the second test proved unreliable, as defined by the first and second test differing (in this case pre-set to $p<0.10$ for main effect of test sequence or for dose by sequence interaction). Once extinction criteria were met, mice were tested for cue-induced reinstatement of alcohol seeking in the two following successive sessions, with sequence of saline vs. exendin-4 presentation counterbalanced.

While first and second reinstatement tests yielded comparable results in the females ($p>0.7$ and no obvious trends), this was not the case in the males (treatment by injection order interaction $p=0.03$). Therefore, only results from the first reinstatement test were used. Order of testing did not significantly affect alcohol self-administration results ($p\geq0.4$ and no obvious trends), which are reported combined below (Supplemental Figure S1).

Oral alcohol self-administration in the Experiment 1 mice

We initially wanted to check, in the same mice used to test cue-induced reinstatement, that exendin-4 decreased alcohol self-administration as has previously been shown, and to test for sex differences in this effect. After reinstatement testing, 20% alcohol solution was again made available as the reinforcer until self-administration returned to baseline levels or met baseline criteria at a new level, and the effect of saline and exendin-4 administration on alcohol self-administration were tested. One male mouse and two female mice that received 3.2 μg/kg exendin-4 and one female mouse mice that received 1.8 μg/kg exendin-4 never re-established alcohol self-administration to criteria levels, and several had increased levels of pokes in the inactive hole. Of the mice that did re-establish alcohol self-administration, males and females took comparable numbers of alcohol reinforcers (29 ± 2.3 and 28.5 ± 1.6, respectively, see Supplemental Figure S1 baseline data indicated as "Base").

Saline and exendin-4 were tested within-subjects, mice that previously received exendin-4 were tested with the same dose, saline mice were pre-assigned one of the doses in counterbalance fashion. Alcohol reinforcers were compared in each sex and dose group by two-way ANOVA with condition (baseline/saline/exendin-4) as repeated-measures factor corrected for cohort.

As shown in Supplemental Figure S1, female mice showed an effect of treatment in the 1.8 μg/kg exendin-4 group [$F(2,30)=11.0$, $p=0.0003$], and in the 3.2 μg/kg group [$F(2,22)=3.70$, $p=0.04$], but responding was decreased relative to baseline after both saline injections ($p=0.02$ and $p=0.002$) and exendin-4 1.8 μg/kg ($p<0.0001$). Alcohol reinforcers taken did not differ significantly between exendin-4 and saline administration at either dose ($p=0.09$, $p=0.3$). Inactive responses tended to go up during re-baseline in the female mice and showed an effect of treatment in the 1.8 μg/kg group ($p=0.02$), with a trend in the 3.2 μg/kg group ($p=0.07$). Post hoc analysis revealed lower inactive responses after 1.8 μg/kg exendin-4 than at baseline ($p=0.02$).

The male mice showed a significant effect of treatment on alcohol reinforcers taken in the 3.2 μg/kg exendin-4 group [$F(2,12)=11.7$, $p=0.002$], but not in the 1.8 μg/kg group ($p>0.5$). The effect in the males reflected decreased alcohol-taking after exendin-4 treatment relative to baseline ($p=0.0004$) and to saline ($p=0.01$), with no significant effect of saline injection relative to baseline. Inactive responses were not significantly affected by treatment in either dose group ($p\geq0.2$).

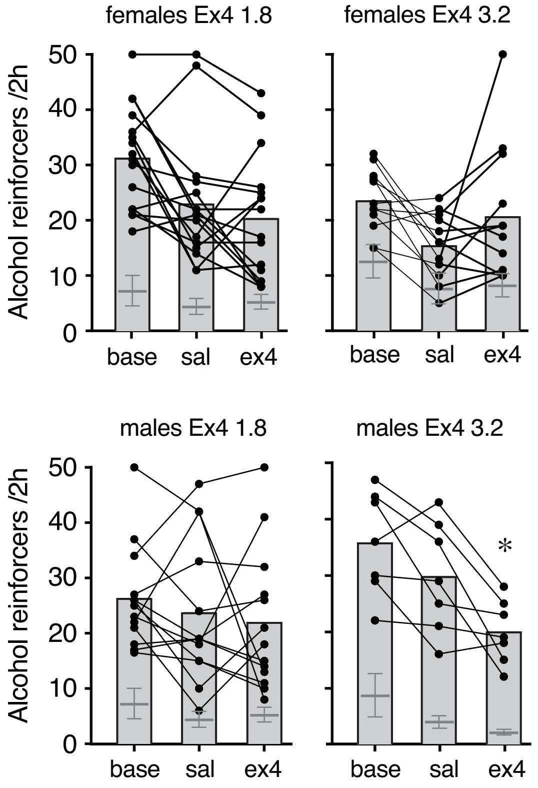

Supplemental Fig. S1 Acute effect of exendin-4 on alcohol self-administration (Experiment 1)
Alcohol reinforcers earned per 2-h session in female (top, n=12-16/dose group) and male (bottom, n=7-13/dose group) mice at re-baseline ("base") and after pretreatment with saline or exendin-4 ("sal", "ex4"). Bars represent group means, dots and lines show all individual mice. Responses made in the inactive nose-poke hole are shown as dark grey lines indicating groups means ±s.e.m. *p=0.01 vs. saline.

Oral alcohol self-administration in the Experiment 2 mice
Baseline self-administration before and after each test suggested a brief (one day) carry-over effect of exendin-4 administration (Fig. S2).

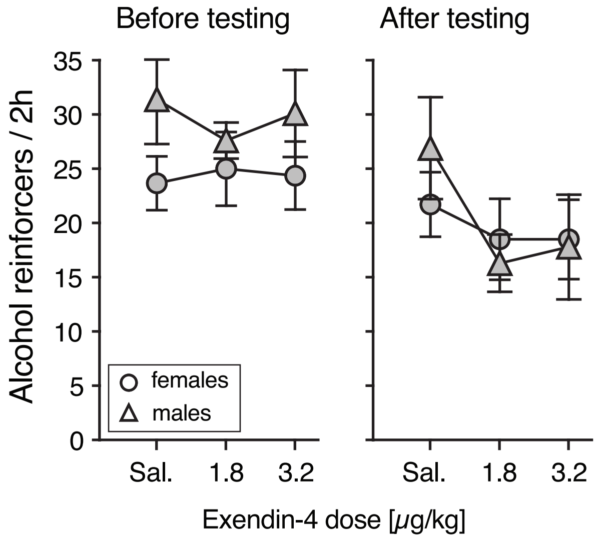

Supplemental Fig. S2 Baseline self-administration on sessions before and after exendin-4 tests (Experiment 2)
Alcohol reinforcers earned per 2-h session in female and male mice on the baseline session immediately before each test session, and on the following baseline session (n=9-10).
